# Effect of three water-regimes on morpho-physiological, biochemical and yield responses of local and foreign olive cultivars under field conditions

**DOI:** 10.1186/s12870-022-03855-8

**Published:** 2022-10-07

**Authors:** Rahmatollah Gholami, Narjes Fahadi Hoveizeh, Seyed Morteza Zahedi, Hojattollah Gholami, Petronia Carillo

**Affiliations:** 1 Crop and Horticultural Science Research Department, Kermanshah Agricultural and Natural Resources Research and Education Center, AREEO, Kermanshah, Iran; 2 Department of Horticultural Science, College of Agriculture, Shahid Chamran University of Ahwaz, Ahwaz, Iran; 3 Department of Horticultural Science, Faculty of Agriculture, University of Maragheh, Maragheh, Iran; 4 Department of Plant Protection, Faculty of Agriculture, University of Kurdistan, Kurdistan, Iran; 5 Department of Environmental, Biological and Pharmaceutical Sciences and Technologies, University of Campania Luigi Vanvitelli, Caserta, Italy

**Keywords:** *Olea europaea.* Drought. Cultivar. Pomological parameters. Osmolyte. Antioxidant enzyme. Oxidative stress marker

## Abstract

**Background:**

Drought stress is among the most serious threats jeopardizing the economic yield of crop plants in Iran. In particular, in response to withholding irrigation, the reduction in performance and quality of a precious plant such as the olive tree is remarkable. Therefore, the selection of cultivars that are resistant or tolerant to drought has been recognized as one of the most effective long-term strategies for sustainably alleviating the adverse effects of this stress. In this view, our study evaluated the response of 8 olive cultivars including 4 elite native cultivars (Zard Aliabad, Roughani, Dezful, and Shengeh) and 4 foreign cultivars (Manzanilla, Sevillana, Konservolia, and Mission) to water shortage in the Dallaho Olive Research station of Sarpole-Zahab in Kermanshah province in 2020. Olive trees underwent 3 levels of irrigation treatment including 100% full irrigation (control), 75%, and 50% deficit irrigation.

**Results:**

Based on the results, 50% deficit irrigation decreased both growth and pomological traits, but determined the highest dry matter percentage. As the severity of drought stress increased, with an accumulation of sodium and malondialdehyde, an incremental increase in osmolytes was observed, as well as an enhancement of the activity of antioxidant enzymes (peroxidase and catalase). In contrast, full irrigation led to an increase in photosynthetic pigments, calcium, and potassium. Dezful and Konservolia cultivars revealed a significantly higher growth rate, correlated in the former to higher levels of chlorophyll, compatible compounds, total phenolic content, relative water content, potassium to sodium ratio, catalase, and peroxidase activities compared with other cultivars. Konservolia showed the best yield parameters under 75% and 100% irrigation regimes, correlated to higher chlorophyll, potassium, and total phenolic content (in particular at 75% ET).

**Conclusions:**

Generally, the selection of more resilient or tolerant cultivars to sustain water scarcity stress is a widely operative solution to extend rainfed orchards in semi-arid environments. Our study showed that Dezful and Konservolia had the best adaptive mechanisms to cope with the detrimental effects of drought stress.

**Supplementary Information:**

The online version contains supplementary material available at 10.1186/s12870-022-03855-8.

## Background

Olive (*Olea europaea* L.) belongs to the family Oleaceae as an evergreen tree and is known as one of the oldest cultivated plants in the world [1]. Globally, olive cultivation has expanded during the last two decades, including olive culture in Iran [2]. Nowadays, due to growing consciousness of the nutritional value of olive, especially olive oil, its market demand and consumption have increased and, as a consequence, its production has expanded. Olive oil is widely known to contain a balanced monounsaturated fatty acids composition, especially oleic acid which can decrease by 30% the risk of the incidence of cardiovascular events. Moreover, olive contains a wide range of micro components such as pentacyclic triterpenes with potential beneficial roles in other pathologies (such as diabetes, obesity, and cancer) [3]. Due to the unique nutraceutical properties of olive oil constituents and the importance of oil quality, the implication of current extreme climate variability for rainfed olive culture and the associated negative impact on olive fruit yield and quality is alarming [4].

Among abiotic stresses, drought is perhaps the main responsible for the damages to agricultural production worldwide, causing approximately 50% yield losses every year globally [5, 6]. The water deficit scenario has repercussions on water relations, nutrient uptake, carbon assimilation, reproduction, and canopy dimension, thus contributing to the decline observed in crop yield and quality [7, 8]. One of the primary deleterious effects caused by drought is the decrease of soil water potential that jeopardizes the ability of plants to absorb water thus decreasing the uptake of nutrients from the soil through the root, and the transport of water to shoot. The consequent water scarcity causes the accumulation of salts in the upper layers of soil further decreasing the capacity of plants to absorb water and nutrient from soil, in particular K^+^ and Ca.^2+^, thus reducing cell turgor pressure [9]. Water stress and disturbance of ions homeostasis do not only impair transpiration causing photosynthetic disfunction but cause overproduction of reactive oxygen species (ROS) and oxidative stress, which damages lipids, nucleic acids, and proteins and destroys the membrane integrity [10]. These negative phenomena determine an exponential decrease in cell division and expansion, and therefore of leaf area, thus inducing early leaf senescence, and abscission, strongly contributing to the decrease in canopy photosynthetic performance [11]

Plants cope with limited moisture supply by inducing different defense adaptation strategies [12]. ‘Yoshhime’ peach demonstrated a high ability of osmotic adjustment, accumulating more sorbitol and proline as compared to glucose, fructose, and sucrose, effectively improving the activities of antioxidant enzymes and enhancing the expression of stress-related genes to counteract lipid peroxidation and limited stomatal conductance [13]. Also, the grapevine responded to drought stress through the mechanisms of regulation of vine water use: decreased stomatal aperture stress and photosynthesis, hydraulic vulnerability segmentation, and leaf osmotic adjustment [14]. In citrus, drought-tolerant rootstocks produced lower amounts of malondialdehyde (MDA) and hydrogen peroxidase (H_2_O_2_), while antioxidant enzymatic activities [superoxide dismutase (SOD), catalase (CAT), and peroxidase (POD)] increased to cope with ROS induced by drought [15]. Five almond cultivars under drought stress reduced plantlet height and the number of developed leaves while increasing proline content, but this was not sufficient to cope with the decrease of relative water content (RWC) and the drought-induced ion leakage [16]. Compartmentalization and some other exclusive molecular mechanisms are other strategies that plants activate to overcome stress conditions [17]. To understand drought stress responses, it is, therefore, essential to unravel the key mechanisms underlying their regulation, and in particular, those depending on growth regulators such as salicylic acid (SA), gibberellins (GAs), cytokinin, and abscisic acid (ABA) [18]. Biosynthesis and accumulation of ABA in the plant tissues are responsible for the activation of several stress-related genes in combination with other hormones such as auxin, cytokinin, and ethylene [19]. To obtain a deeper insight into various genes involved in plant development under drought stress, the functional characterization of the Arabidopsis transcription factor (TF) bZIP29 revealed its role in leaf and root development also linked to other hormones signaling crosstalk [20], such as those of auxin, ethylene and ABA [21]. Moreover, the *RAP2.12* gene as an ethylene responsive TF participates in several hormone-signaling pathway including those of jasmonic acid, SA, and ethylene, and ultimately plays a role in drought tolerance [22]. The ability of exposition to abiotic stresses is related to high variability of the species for stress tolerance and characterizing of the genetic determines controlling plant response to the stress and candidate genes involved to drought stress tolerance [23].

Although in extreme drought stress, olive displays different levels of drought tolerance in the predicted scenarios, considerable productive parameters compromising fruit yield, quality, and oil manufacturing in olive are damaged [4]. Three layers of cylindrical cells are in palisade-like parenchyma. In this kind of parenchyma, intercellular spaces are smaller than those of the spongy parenchyma [24]. These exclusive leaf anatomical traits are more suitable to optimize the internal conductance of water vapor transport. Additionally, olive leaves are covered with a thick cuticle, waxy layer, and trichomes hiding the small and abundant stomata to provide diffusional limitations [8]. Small stomata in olive leaves are spread only on the abaxial surface (hypostomatic), being even smaller and denser in response to a shortage of water, thus controlling water use efficiency. Olive trees also largely benefit from hydraulic redistribution, the ability of roots to penetrate deeper soil layers to better uptake water, in line with the detrimental effect of drought on the upper dry soil layers. On the other hand, the olive tree is characterized by a strong capacity for osmotic adjustment; in fact, it can accumulate compatible solutes thus increasing osmotic potential to promote a soil–plant water gradient, which can extract water from the soil potentially even below the wilting point. To survive the toxic effect of ROS, the olive tree has evolved an efficient antioxidant defense system. Meanwhile, under severe drought conditions, ROS production often increases lipid peroxidation, MDA production, and DNA and protein degradation [25]. Plant resistance to water deficit has been correlated to a higher net photosynthetic rate and higher capacity for osmotic adjustment with proline in the ‘Chemlali’ olive cultivar [26]. So, depending on the genotype, olive cultivars manifest different levels of water deficit tolerance [4].

Indeed, drought is the most acute abiotic stress in Iran and many other countries of the world; this issue directly reduces the chance for olive cultivation as a potentially profitable system. Therefore, it is important to make olive cultivation more sustainable under severe drought, thanks to the selection of more tolerant cultivars according to necessities in arid and semi-arid regions. Due to the chance to categorize higher drought-tolerant olive genotypes, it is possible to introduce and compare other new genotypes and cultivars with higher levels of stress tolerance [27]. In particular, we examined and evaluated the tolerance of cultivars adapted to the Iranian climate (elite native cultivars) in comparison with foreign olive cultivars under three irrigation regimes to study their morphological, physiological, and biochemical traits and recognize the most drought-tolerant cultivar. Despite the numerous studies on drought stress effects on olive plants, studies on twenty years old olive plants in a field are scarce and the response mechanisms to water deficit are still not fully elucidated and claimed for investigation. The identification of the most tolerant cultivars can be exploited both for future more in-depth studies on the molecular mechanisms underlying drought tolerance and to design new agronomic strategies for olive cultivation to be translated directly into the field to improve oil production even under stress conditions.

## Results

### Current season, shoot growth and diameter and fruit weight, length, diameter, and dry matter

Cultivars and irrigation treatments had important effects on all growth and pomological characteristics separately; however, their interaction effect was not statistically significant (Table 1). Current season shoot growth varied between Dezful (19.99 cm) and Sevillana (13.26 cm), whereas shoot diameter was highest in the cultivar Konservolia (0.36 cm) and lowest in the Roughani one (0.24 cm). Konservolia exhibited significantly higher fresh fruit weight compared to all other cultivars (5.55 g), whereas the Mission cultivar produced the lowest average fruit weight (2.76 g). Among the investigated olive cultivars, fruit length ranged from 2.60 cm (Dezful) to 2.01 cm (Sevillana) and fruit diameter ranged from 2.01 cm (Konservolia) to 1.42 cm (Sevillana). Therefore, Dezful exhibited significantly longer fruits, while Konservolia produced fruits of significantly larger diameter than the other investigated olive cultivars. According to the obtained results, the highest and lowest dry matter was observed in Roughani (44.96%) and Manzanilla (32.76%), respectively.

**Table 1:** The effect of different cultivars and irrigation regimes on current season shoot growth and diameter, fruit weight, length and diameter and dry matter of eight elite native and foreign olives

Source of Variance	Current season shoot growth	Current season shoot diameter	Fruit weight	Fruit length	Fruit diameter	Dry matter
	**(cm)**		**(g)**	**(cm)**		**(%)**
**Cultivar**						
Manzanilla	15.34 ± 0.27d	0.26 ± 0.01cd	3.96 ± 0.13bc	2.09 ± 0.03de	1.62 ± 0.07c	32.67 ± 1.25f
Sevillana	13.26 ± 0.16e	0.25 ± 0.03cd	2.96 ± 0.13de	2.01 ± 0.05e	1.42 ± 0.07e	35.46 ± 1.47d
Mission	17.34 ± 0.27c	0.29 ± 0.01bc	2.76 ± 0.13e	2.09 ± 0.03de	1.52 ± 0.07d	35.46 ± 1.47d
Zard ali abad	14.30 ± 1.05de	0.28 ± 0.02bcd	3.33 ± 0.17d	2.13 ± 0.06d	1.58 ± 0.03cd	37.87 ± 1.20c
Roughani	13.40 ± 1.01e	0.24 ± 0.02d	2.96 ± 0.15de	2.06 ± 0.06de	1.51 ± 0.02d	44.96 ± 0.68a
Konservolia	19.32 ± 1.36ab	0.36 ± 0.04a	5.55 ± 0.12a	2.41 ± 0.03c	2.01 ± 0.01a	34.06 ± 1.52e
Dezful	19.99 ± 0.97a	0.30 ± 0.05b	4.15 ± 0.37b	2.60 ± 0.03a	1.74 ± 0.04b	34.06 ± 1.52e
Shengeh	18.47 ± 0.70bc	0.30 ± 0.03b	3.74 ± 0.03c	2.51 ± 0.03b	1.62 ± 0.01c	41.04 ± 0.27b
**Irrigation (%)**						
100	19.19 ± 0.52a	0.36 ± 0.01a	4.25 ± 0.11a	2.40 ± 0.03a	1.73 ± 0.07a	33.94 ± 1.13c
75	16.33 ± 0.48b	0.28 ± 0.01b	3.63 ± 0.09b	2.24 ± 0.04b	1.62 ± 0.02b	37.35 ± 0.75b
50	13.76 ± 0.56c	0.21 ± 0.01c	3.14 ± 0.17c	2.08 ± 0.01c	1.53 ± 0.03c	39.55 ± 1.25a
*Cultivar*	**	**	**	**	**	**
*Irrigation*	**	**	**	**	**	**
Cultivar*Irrigation	*ns*	*ns*	*ns*	*ns*	*ns*	*ns*
*LSD*	*0.75*	*0.03*	*0.24*	*0.05*	*0.04*	*0.81*

ns, ** Non-significant or significant at *p* < 0.01. Different letters within each column indicate significant differences according to LSD multiple range test (*p* = 0.05)

The irrigation regime significantly affected the evaluated growth parameters. Current season shoot growth and shoot diameter, as well as fruit weight, length, and diameter significantly decreased with increasing levels of drought stress. Conversely, the fruit dry matter increased significantly as the irrigation deficit level increased, ranging from 33.94% under full irrigation to 39.55% at 50% deficit irrigation (Table 1).

### Photosynthetic pigments

While photosynthetic pigment levels were significantly influenced by irrigation and cultivar treatments, the cultivar x irrigation treatment interaction was found non-significant (Table 2). As a result, the highest levels of chlorophyll a (Chl a), chlorophyll b (Chl b), and total chlorophyll (total Chl) were found in the Dezful cultivar (0.96, 0.48, and 1.45 mg g^−1^ fresh weight (FW), respectively), as compared to Sevillana (0.44, 0.16 and 0.68 mg g^−1^ FW, respectively) that showed the lowest levels (Table 2). The content of photosynthetic pigments decreased as the water deficit increased, with a reduction of 49, 36, and 65% for Chl a, Chl b, and total Chl, respectively, from 100% full irrigation presented to 50% deficit irrigation (Table 2).

**Table 2:** The effect of different cultivars and irrigation regimes on Chl a, Chl b and total Chl of eight elite native and foreign olives

Source of Variance	Chl a	Chl b	Total Chl
	**(mg g**^**−1**^**FW)**		
**Cultivar**			
Manzanilla	0.50 ± 0.04cd	0.20 ± 0.02de	0.82 ± 0.04d
Sevillana	0.44 ± 0.04d	0.16 ± 0.01e	0.68 ± 0.03e
Mission	0.56 ± 0.11c	0.24 ± 0.02 cd	0.90 ± 0.14cd
Zard ali abad	0.58 ± 0.05c	0.27 ± 0.04c	0.94 ± 0.09c
Roughani	0.45 ± 0.06d	0.17 ± 0.10e	0.72 ± 0.07e
Konservolia	0.86 ± 0.09b	0.46 ± 0.06a	1.43 ± 0.04a
Dezful	0.96 ± 0.03a	0.48 ± 0.04a	1.45 ± 0.01a
Shengeh	0.81 ± 0.06b	0.36 ± 0.03b	1.35 ± 0.01b
**Irrigation (%)**			
100	0.84 ± 0.04a	0.36 ± 0.02a	1.58 ± 0.06a
75	0.66 ± 0.04b	0.29 ± 0.02b	0.98 ± 0.05b
50	0.43 ± 0.05c	0.23 ± 0.02c	0.55 ± 0.02c
*Cultivar*	**	**	**
*Irrigation*	**	**	**
Cultivar*Irrigation	*ns*	*ns*	*ns*
*LSD*	*0.05*	*0.04*	*0.05*

Means having the same letter in traits are not significantly different by LSD multiple range test at 5%

ns, ** Non-significant or significant at *p* < 0.01. Different letters within each column indicate significant differences according to LSD multiple range test (*p* = 0.05)

### Osmolytes (soluble carbohydrate content, proline content, and TPC) and RWC

Osmolytes and RWC were affected by irrigation and type of cultivar but no significant interaction was reported between them (Table 3). Dezful and Sevillana showed the highest (13.26 mg g^−1^ FW) and lowest (8.88 mg g^−1^ FW) contents of soluble carbohydrates, respectively. Dezful also showed the highest levels of proline (40.83 µg g^−1^ FW), TPC (152.17 mg 100 g^−1^ FW), and RWC (77.06%) among the examined cultivars. Roughani exhibited the lowest amounts of proline (34.38 µg g^−1^ FW) and RWC (59.45%), while Sevillana showed the lowest contents in TPC (75.78 mg 100 g^−1^ FW) among the tested cultivars. The levels of soluble carbohydrates, proline, and TPC, averaged over cultivars, increased significantly as drought levels increased while RWC decreased significantly with the increase of drought stress (Table 3).

**Table 3:** The effect of different cultivars and irrigation regimes on soluble carbohydrate content, proline content, total phenolic content (TPC) and relative water content (RWC) of eight elite native and foreign olives

Source of Variance	Soluble carbohydrate content	Proline content	TPC	RWC
	**(mg g**^**−1**^**FW)**	**(µg g**^**−1**^**FW)**	**(mg 100 g**^**−1**^**FW)**	**(%)**
**Cultivar**				
Manzanilla	9.89 ± 0.78cd	37.89 ± 1.10bc	110.77 ± 9.92c	69.06 ± 1.59c
Sevillana	8.88 ± 1.04e	35.33 ± 2.62de	75.78 ± 26.65d	60.83 ± 1.36d
Mission	10.62 ± 1.01bc	35.75 ± 1.76cde	116.38 ± 33.44bc	72.51 ± 1.54b
Zard ali abad	10.38 ± 0.85bc	37.05 ± 0.76cd	112.72 ± 11.41bc	69.28 ± 1.39c
Roughani	8.94 ± 0.71de	34.38 ± 0.41e	79.00 ± 28.09d	59.45 ± 0.61d
Konservolia	12.49 ± 1.01a	40.28 ± 1.69a	151.78 ± 13.16a	76.67 ± 1.61a
Dezful	13.26 ± 0.69a	40.83 ± 1.11a	152.17 ± 16.47a	77.06 ± 0.57a
Shengeh	11.23 ± 0.72b	39.88 ± 1.21ab	131.40 ± 12.38b	72.67 ± 1.62b
**Irrigation (%)**				
100	6.64 ± 0.09c	14.09 ± 1.10c	91.55 ± 17.48c	80.65 ± 0.49a
75	10.10 ± 0.97b	36.18 ± 0.74b	121.06 ± 12.12b	68.28 ± 0.46b
50	15.40 ± 0.73a	62.75 ± 0.24a	136.14 ± 11.32a	60.14 ± 1.35c
*Cultivar*	**	**	**	**
*Irrigation*	**	**	**	**
Cultivar*Irrigation	*ns*	*ns*	*ns*	*ns*
*LSD*	*0.61*	*1.37*	*12.37*	*1.29*

Means having the same letter in traits are not significantly different by LSD multiple range test at 5%

ns, ** Non-significant or significant at *p* < 0.01. Different letters within each column indicate significant differences according to LSD multiple range test (*p* = 0.05)

### Mineral elements: calcium (Ca), potassium (K), and sodium (Na)

Cultivar and irrigation regimes were both highly significant regarding contents of Ca, K, and Na, but there was no significant interaction between treatments regarding the levels of these elements (Table 4). The highest accumulation of sodium occurred in Roughani [0.36% dry weight (DW)] and the minimum amount was observed in Dezful (0.19% DW). Dezful exhibited the highest amounts of Ca and K (2.42% and 1.53% DW, respectively) Sevillana and Roughani showed the lowest values of Ca (1.62% DW), while Roughani alone had the lowest K content (0.92% DW) among the examined olive cultivars (Table 4).

**Table 4:** The effect of different cultivars and irrigation regimes on sodium (Na), calcium (Ca) and potassium (K) of eight elite native and foreign olives

Source of Variance	Na	Ca	K
	**(% DW)**		
**Cultivar**			
Manzanilla	0.29 ± 0.03bc	1.95 ± 0.14b	1.10 ± 0.14de
Sevillana	0.35 ± 0.05a	1.62 ± 0.44c	1.00 ± 0.11ef
Mission	0.26 ± 0.03 cd	2.16 ± 0.28ab	1.45 ± 0.07ab
Zard ali abad	0.32 ± 0.03ab	2.17 ± 0.07ab	1.20 ± 0.04cd
Roughani	0.36 ± 0.01a	1.62 ± 0.08c	0.92 ± 0.08f
Konservolia	0.20 ± 0.05e	2.32 ± 0.33a	1.50 ± 0.11a
Dezful	0.19 ± 0.01e	2.42 ± 0.18a	1.53 ± 0.11a
Shengeh	0.24 ± 0.01d	2.20 ± 0.21ab	1.33 ± 0.11bc
**Irrigation (%)**			
100	0.18 ± 0.01c	2.88 ± 0.2a	1.45 ± 0.1a
75	0.26 ± 0.02b	1.96 ± 0.14b	1.27 ± 0.05b
50	0.38 ± 0.02a	1.34 ± 0.24c	1.05 ± 0.05c
*Cultivar*	**	**	**
*Irrigation*	**	**	**
Cultivar*Irrigation	*ns*	*ns*	*ns*
*LSD*	*0.02*	*0.18*	*0.09*

Means having the same letter in traits are not significantly different by LSD multiple range test at 5%

ns, ** Non-significant or significant at *p* < 0.01. Different letters within each column indicate significant differences according to LSD multiple range test (*p* = 0.05)

Across cultivars, the levels of Na increased significantly as drought stress increased, reaching 0.38% DW at the 50% deficit level. The highest levels of Ca and K were present in the 100% irrigation treatment, achieving levels of 2.15-and 1.38-fold higher than the respective Ca and K levels observed in the 50% deficit irrigation (Table 4).

### Enzymes (CAT and POD) and marker (MDA)

There were significant effects of cultivar and irrigation regime on oxidative enzymes and the marker MDA, but there was no significant interaction effect between these variables (Table 5). CAT activity varied significantly among cultivars, ranging from 1.24 units mg^−1^ (Dezful) to 0.76 units mg^−1^ (Sevillana). Dezful showed also the highest POD activity (3.02 units mg^−1^) among cultivars. MDA content ranged from 3.69 nmol g^−1^ FW (Sevillana) to 2.13 nmol g^−1^ FW (Dezful), varying significantly among cultivars. Across cultivars, the activities of the two oxidative enzymes and MDA increased significantly as drought stress increased. CAT activity under 50% irrigation treatment was observed to be nearly threefold higher than in trees with full irrigation. Whereas both POD activity and MDA content almost doubled in trees irrigated at a 50% deficit compared to those with full irrigation.

**Table 5:** The effect of different cultivars and irrigation regimes on catalase (CAT), peroxidase (POD) activity and malondialdehyde (MDA) content of eight elite native and foreign olives

Source of Variance	CAT	POD	MDA
	**(units mg**^**−1**^**)**		**(nmol g**^**−1**^**FW)**
**Cultivar**			
Manzanilla	0.90 ± 0.05c	2.41 ± 0.26c	3.35 ± 0.17ab
Sevillana	0.76 ± 0.01d	1.70 ± 0.14d	3.69 ± 0.58a
Mission	1.02 ± 0.04b	2.65 ± 0.11bc	2.88 ± 0.62c
Zard ali abad	0.95 ± 0.05c	2.65 ± 0.13bc	3.21 ± 0.13bc
Roughani	0.78 ± 0.04d	1.94 ± 0.39d	3.56 ± 0.59ab
Konservolia	1.23 ± 0.05a	2.99 ± 0.17a	2.15 ± 0.26d
Dezful	1.24 ± 0.02a	3.02 ± 0.05a	2.13 ± 0.53d
Shengeh	1.07 ± 0.01b	2.83 ± 0.04ab	2.24 ± 0.52d
**Irrigation (%)**			
100	0.57 ± 0.01c	1.46 ± 0.08c	2.07 ± 0.19c
75	0.92 ± 0.03b	2.45 ± 0.14b	2.70 ± 0.34b
50	1.49 ± 0.03a	3.66 ± 0.08a	3.94 ± 0.43a
*Cultivar*	**	**	**
*Irrigation*	**	**	**
Cultivar*Irrigation	*ns*	*ns*	*ns*
*LSD*	*0.04*	*0.18*	*0.46*

Means having the same letter in traits are not significantly different by LSD multiple range test at 5%

ns, ** Non-significant or significant at *p* < 0.01. Different letters within each column indicate significant differences according to LSD multiple range test (*p* = 0.05)

### Yield parameters and water use efficiency

**Fig. 1:**
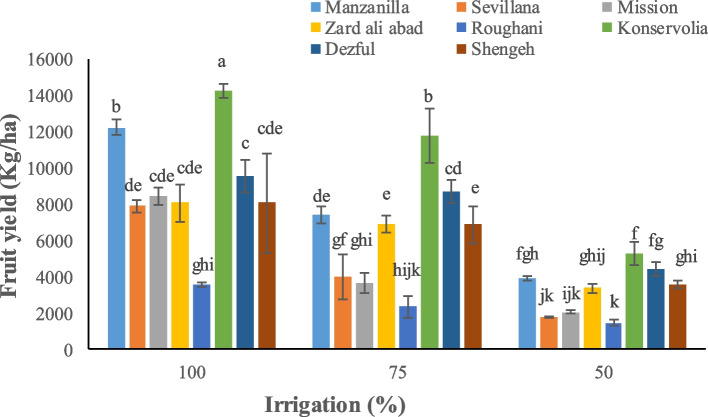
The effect of different cultivars and irrigation regimes on fruit yield of eight elite native and foreign olives

**Fig. 2:**
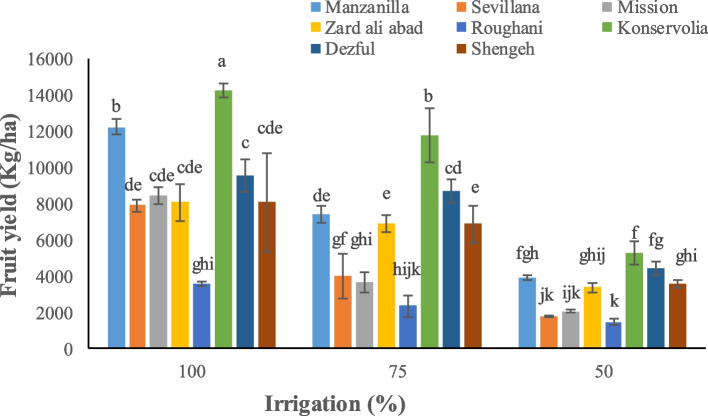
The effect of different cultivars and irrigation regimes on oil yield of eight elite native and foreign olives

**Fig. 3:**
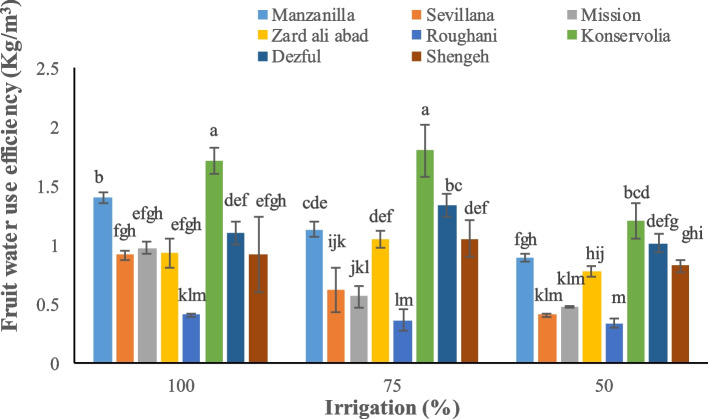
The effect of different cultivars and irrigation regimes on fruit water use efficiency of eight elite native and foreign olives

Irrigation and type of cultivar significantly affected yield parameters. Konservolia showed the highest fruit yield both under 100% and 50% irrigation treatment (14875.5 and 5231.3 kg ha^−1^, respectively), while Rughani showed the lowest fruit yield both under 100% and 50% irrigation ones (3518.4 and 1435.1 kg ha^−1^, respectively) (Fig. 1). Konservolia among all cultivars showed the highest oil yield of 1879.8 lit ha^−1^ under 100% irrigation whereas Mission, under 50% deficit irrigation, presented the lowest oil yield (147.3 lit ha^−1^) (Fig. 2). Konservolia also exhibited the highest water use efficiency in the 75% deficit irrigation (1.8 kg m^−3^) while cultivar Rughani showed the lowest water use efficiency under 50% deficit irrigation (0.33 kg m^−3^) (Fig. 3).

**Fig. 4:**
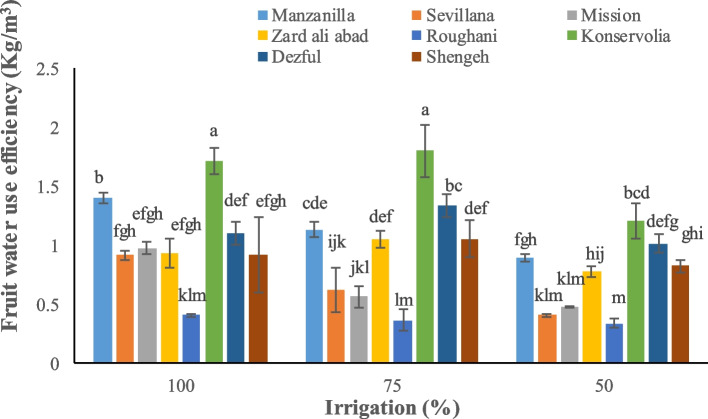
The effect of different cultivars and irrigation regimes on fresh oil content of eight elite native and foreign olives

According to the obtained results, the highest accumulation of oil content occurred in the cultivar Roughani (17.50% FW) under 75% irrigation while the minimum amount was observed in the cultivar Mission (6.14% FW) under 100% irrigation treatment (Fig. 4).

### Principal component analysis (PCA)

**Fig. 5:**
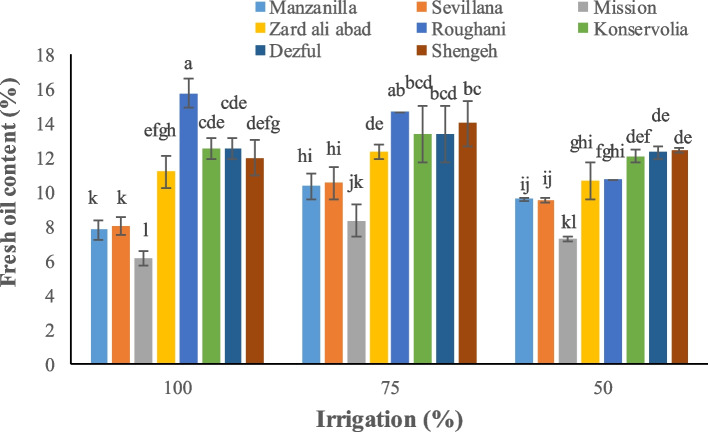
Principal component analysis (PCA) of morphological, biochemical and physiological traits in the investigated olive cultivars grown under 100, 75 and 50% irrigation treatments

Treatments and cultivars were separated by applying a principal component analysis (PCA). The first two principal components (PCs) were related with eigenvalues higher than 1 and explained 84.1% of the total variance, with PC1and PC2 accounting for 64.3% and 19.8%, respectively (Fig. 5). The irrigation regimes contributed to the clear separation on PC1, whereas the cultivars contributed to the separation on PC2. The cultivars Konservolia, Dezful, and Shengeh treatments under a 100% irrigation regime were clustered on the positive side of PC1 and correlated to photosynthetic pigments, RWC, shoot growth and diameter, Ca, K fruit, and oil yield. Whereas Roughani and Sevillana under 50% irrigation were on the negative side of PC1 and correlated with Na, MDA, ion exchange, and proline. The cultivars Konservolia and Dezful were clustered on the positive side of PC2 and correlated with total phenolic content (TPC), antioxidant enzyme activities (CAT and POD), and soluble carbohydrates; while Roughani and Sevillana under 100% irrigation were on the negative side of PC2 and slightly correlated to water use (Fig. 5).

## Discussion

Water deficit is one of the most important edaphic stresses affecting plant growth and development and the most significant factor that limits olive yield [8]. The selection of drought-resistant olive lines provides an important reference tool for improving the efficiency of production concerning water consumption.

### Growth and pomological parameters

As demonstrated in this study, Dezful showed the largest current season shoot growth and fruit length; Konservolia, whose current season shoot growth did not differ significantly from Dezful, showed the highest current season shoot diameter, as well as fruit weight and diameter. Further, Roughani exhibited the highest percentage of dry matter. The 50% deficit irrigation decreased both growth and pomological parameters in all cultivars, with a particularly strong effect on the cultivar Mission, which is particularly sensitive to irrigation regime as previously demonstrated by [28]. Indeed, the vegetative characteristics of olive trees are correlated with the genotype and the irrigation water regimes, since the amount of received water can affect the vegetative growth of olive trees. In particular, the development of vegetative traits (e.g., height, crown size, and trunk diameter), is jeopardized by the restriction of irrigation in all cultivars [29]. The decrease in RWC caused an incremental increase in the dry matter during a water shortage, in particular in the Roughani cultivar [30]. In another study, water shortage inhibited biomass accumulation more in roots than in shoots [31].

The restriction and/or interruption of water flow from the xylem to the surrounding cells determines water shortage and consequently a loss of turgor that affects the rate of cell expansion and division, cell elongation, organ expansion, and therefore growth. Stem diameter growth rate is an indicator of both plant growth and water status [32]. This is consistent with the findings of the present research. Considerably, if a high water deficit occurs in the most sensitive phases of vegetative growth in olive trees, stressful conditions highly affect olive production [33]. Drought stress due to the detrimental effect on cell division, differentiation, and enlargement, can reduce plant growth [34]. These decreases in growth parameters may be related to the decline of RWC and shrinkage of cells, decrease of leaf growth as well as cell division, leaf production blockage, senescence acceleration (due to ABA accumulation), and ultimately leaf drop [35]. Moreover, drought stress can indirectly reduce the import of carbon dioxide into stomata when exposed to water stress and photosynthesis is restricted due to stomata closure, with a consequent decrease of vegetative growth [36].

### Photosynthetic pigments

The highest values of Chl a, Chl b, and total Chl have been recorded in Dezful cultivar under 100% full irrigation. Severe drought stress (50% deficit irrigation) strongly decreased photosynthetic pigments, in particular, Chl a, and b, as also previously shown in olive trees [37] as well as in marigold cultivars [38]. Relative decrease in Chl a content and efficiency of photosystem ΙΙ are effective adaptive strategies for drought tolerance [39]. Water shortage elicits a strong stomatal closure as the earliest response with a consequent decrease of carboxylation capacity of photosynthesis for a decrease in CO_2_ availability [40]. This leads to a progressive accumulation of NADPH and ATP, which ultimately results in downregulation or feedback inhibition of the photosynthetic electron transport [41].

In these conditions, there is a reduction in Chls synthesis as a photoprotective mechanism; in fact, the Chl loss reduces the amounts of photons absorbed via leaves allowing the leaf tissues to reduce photooxidation and overcome the severe water stressed period [33, 42]. Chl loss and pigment photooxidation are considered obvious symptoms of oxidative stress as a consequence of water stress. Based on this fact, preserving a high level of antioxidative enzyme activities, and enhancing the capacity of host plants against oxidative damage can contribute greatly to drought stress alleviation [43].

In the study of Calvo-Polanco et al. [44], the drought treatment induced a significant reduction in the leaf Chl content of olive trees. These pigments are crucial components for Photosystems II and I and light-harvesting complexes (LHC), and oxidative stress can cause their photo-oxidation and degradation, affecting photosynthesis more than the restriction of CO_2_ caused by stomatal closure during water deficit [45, 46]. However, the drought stress-related restriction to CO_2_ uptake caused by leaf stomata closure varies among plant species, so drought tolerance depends on the cultivar [47]. In rice, genes like chlorophyll a/b-binding protein CP24, PSI reaction center subunit V, protochlorophyllide reductase A and peptidyl-prolyl cis–trans isomerase, involved in photosynthesis, are down-regulated in response to drought stress [48]. In chickpea cultivars, drought stress equally affected chl a and b [49]. During Chl degradation under drought stress, α-tocopherol, an antioxidant involved in the O^−^_2_ scavenging, can be synthesized through the phytol recycling pathway. The accumulation of this photoprotective molecule with a decline of Chl content is an effective strategy for highly drought-tolerant plants to survive [50]. Chl a under drought stress could be degraded more compared to Chl b, indicating to be a more sensitive photosynthetic pigment prone to degradation to decrease the amount of excitation energy reaching chl a at the reaction center, and the electron transfer to an impaired electron transport chain under stress. The decrease in Chl a content and efficiency of photosystem ΙΙ could be adaptive strategies for drought tolerance [39]. However, chlorophyll degradation occurs from Chl a, while to degrade Chl b, it must be first converted to Chl a by two sequential enzymes, Chl b reductase and hydroxyl methyl Chl a reductase (HMCR). Therefore, if the transcription or activity of these two enzymes is decreased by drought stress, Chl b accumulates compared to Chl a [51].

### Osmolytes, phenolics, and RWC

Dezful and Konservolia showed the highest osmolyte content and RWC among all cultivars. At more advanced drought stages, total carbohydrates increased in all cultivars differently from those found in Passulunara and Biancolilla Siracusana olive cultivars in previous studies [27]. Soluble carbohydrates (such as glucose, fructose, and sucrose), as well as amino acids such as proline, are the major solutes supporting osmotic adjustment in olive trees [26, 52] as well as in other plants under different environmental stresses [53–55]. Plant growth mainly depends upon storage carbohydrates especially soluble sugars as a mobilized form [56]. Under drought stress, the accumulation of soluble carbohydrates as an osmotic adjustment is able to decrease the water potential of the cells to increase and/or maintain water influx and assist in maintaining tissue turgor. Mannitol and sucrose as main photosynthetic products to transfer in the phloem in the long distances, were induced a stronger accumulation in water stressed compared to well watering olive plants. The accumulation of osmoprotectants such as sugar and phenolic compounds is an initial mechanism to induce enhancement of resistance in olive to drought stress [31]. Proline is one of the most widely distributed compatible compounds, which accumulates in plants under abiotic stress conditions [57]. The increase of proline levels as an osmoprotectant may facilitate water retention, and are considered as an adaptive mechanism [58]. Accumulation of proline in the leaves under acute water deficit was observed in several olive genotypes [59]. It is not clear if its accumulation in tissues under dehydration is a stress symptom, a stress response, or an adaptative strategy. However, in addition, acting as an osmolyte to balance the decrease in water potential, proline can buffer cellular redox potential, detoxify ROS and stabilize macromolecules, thus reducing cell and tissue damages [60, 61]. To provide a level of resistance to drought stress, plants activate several mechanisms such as increasing the accumulation of certain osmolytes. For example, proline can also play a key role in ROS scavenging [62]. It, even being an amino acid accumulated under osmotic stress as an osmolyte, has been found able to act as a ROS scavenger, protect and stabilize membranes and macromolecules, and promote the expression of stress-responsive genes presenting elements responsive to proline [9].

The fact that the plants have developed systems relying upon low molecular weight substances, such as proline or phenolics, highlight the use of common but highly efficient components and pathways in plant response to related stresses. As an example, phenolic compounds are natural secondary metabolites, based on their special chemical structures, possess noticeable antioxidant properties [25]. Their increase was commonly reported in olive plants under water deficit [63]. In particular, 75% and 50% deficit irrigation boosted levels of phenolics while 100% deficit irrigation decreased it in all cultivars, as also previously reported by [4]. Phenolic compounds play an important antioxidative role by participating in several mechanism as free radicals scavengers, peroxidase enzyme substrates, oxidative and oxygen reactions’ blockers, and metal ion chelators [64].

In the studied olive trees, the highest RWC was obtained under 100% full irrigation in Konservolia and Roughani while the minimum RWC was observed in Sevillana in response to severe water stress (50% deficit irrigation). In all cultivars, deficit irrigation caused a decrease in RWC as also reported by Elhami et al. [59]. Mechri et al. [31] proved that unstressed olive plants indicated maintained RWC levels (85%), but this trait reduced to 52% in plants exposed to water deficit. Resistant cultivars preserve their RWC at a higher drought stress level than susceptible ones under similar conditions [65]. Therefore, since RWC remains higher in the leaves of plants that better tolerate drought stress; thus being widely utilized as an authentic index for screening drought-tolerant cultivars.

### Mineral elements

In this study, Dezful showed the highest levels of Ca under 100% full irrigation and 75% deficit irrigation (not shown), while Sevillana and Roughani were the lowest ones. Konservolia and Dezful showed also the highest and lowest levels of K and Na, respectively, whereas the contrary happened for Sevillana and Roughani cultivars. Brito et al. [8] suggested that the lower amounts of Ca and K and the higher amount of Na under drought stress could be related to drought stress susceptibility. In Sevillana and Roughani cultivars, Ca and K content of the leaves decreased in the treatments under drought stress while Na was enhanced. In a soil moisture deficit situation, like that induced in our experiment under 75% and 50% water deficit, the solubility, and transportation rate of Ca from soil to root surface decreases. This can be due both to the decrease of soil water potential and the fact that under drought stress situations, colloids in the soil absorb K more powerfully and prevent its uptake by the roots. Moreover, drought decreases the synthesis of transport proteins involved in the uptake and transport of nutrients [66]. So, drought stress can avert the absorption, transport, and subsequent distribution of minerals in the plant [8].

### Oxidative enzymes and markers

The mechanisms involved in the plant reaction to induced water limitation enhance the antioxidative enzymatic activities [67]. Dezful and Konservolia cultivars showed the largest CAT and POD enzymatic activities and lowest MDA content, while the opposite happened for the cultivar Sevillana which showed the lowest antioxidant enzymatic activities but the highest MDA. Gholami and Zahedi [4] found that the highest amount of MDA was observed in olive cultivars under 50% deficit irrigation. MDA is one of the final products of polyunsaturated fatty acids peroxidation by ROS in the cells and is therefore indicated as an index of the level of membrane lipid peroxidation in plants under stress. Previous studies demonstrated that the accumulation of MDA increased significantly in response to drought stress [33]. Elhami et al. [59] demonstrated that a 40% water deficit in olive plants enhanced POD activity compared with full irrigation treatment (100% FC). Moreover, changes in the activities of CAT and POD due to abiotic stresses have been previously shown in olive crops [63]. The enhancement in antioxidative activity and metabolites was reported also in another research on olive trees under water deficit [68]. Under severe stress conditions, an imbalance between ROS synthesis and the antioxidant defense system may occur [7] thus causing an accumulation of ROS, lipid peroxidation, and cell damage, with repercussions on plant growth and development, and finally yield performance [69]. For adjusting the balance by detoxification of excess ROS, the enhancement in activities of antioxidant enzymes (POD and CAT) is required [33, 70]. In general, to induce ROS scavenging under drought stress, plants develop a complicated antioxidant defensive strategy [71]. CAT is essential to assimilate and detoxify H_2_O_2_ in peroxisomes [13] and POD is also in charge of the H_2_O_2_ decomposition [72]. In a study between 6 different citrus rootstocks, tolerant rootstocks exhibited less MDA and H_2_O_2_ but higher activities of antioxidant enzymes (CAT and POD) to cope with ROS [15]. However, the fine tuning of ROS scavenging enzymes and antioxidant system can also allow to maintain a beneficial low ROS concentration able to play a key role in ROS-hormones integrated signal events triggering stress-specific defense or tolerance responses. In particular, a controlled ROS increase may be linked to the drought perception/sensing and activation of i) ABA and other hormones, ii) homologs of respiratory burst oxidase homolog (RBOH) and iii) calcium fluxes via ABA-dependent or independent signaling pathways [73]. In fact, ROS can activate a positive feedback loop involving ABA and resulting in higher ROS/ABA levels able to modulate gene expression and cellular responses to cope with drought stress [74]. ABA-induced transcription factors (TFs) may also play an important role in promoting drought tolerance through ROS signaling. In Arabidopsis, for example, Redox Responsive Transcription Factor 1 (RRTF1) belonging to the APETALA 2/ethylene-responsive element binding factor (AP2/ERF) family, is a component of the central redox signaling network, stimulated by ABA and ROS in response to various stresses, including drought [75]. Similarly to ABA, brassinosteroids (BR) have been found able to boost the transcription of Respiratory Burst Oxidase Homolog1 (RBOH1) and the activity of NADPH oxidase thus increasing the concentration of apoplastic H_*2*_O_*2*_ under drought [76, 77]. The increase of apoplastic ROS can activate the ROS/calcium‐activated calcium channels, promoting calcium fluxes which boost ROS production by RBOH in the neighbouring cells and tissues and generating a “ROS wave” responsible for the activation of systemically acquired acclimation (SAA) in plant tissues [78]. Salicylic acid (SA) can also play a role in promoting the accumulation of ABA while antagonising auxin-signaling pathway, thus functioning as a further signal for the development of the SAA ([79] and references therein). In particular, in olive trees, the adaptability to recurrent drought episodes mediated by SA is achieved by improving the balance between ROS production and scavenging, the plant ionome regulation, and promoting root development [80]. The same effect on root enlargement can be exerted by other phytohormones, that can have a crosstalk with ROS playing a decisive role to allow plants to adapt to drought. In fact, ROS accumulation can reduce auxin and CK accumulation and/or signaling, altering plant shoot growth in order to enlarge root while reducing the surface of evapotranspirating organs, lowering stomatal density and/or conductance [81, 82]. In, particular the CK signaling under drought stress can be controlled by ABA by both indirect and direct mechanisms. The ABA indirect regulation of CK is exerted by mean of ABA-responsive myelocytomatosis oncogenes (MYC) and dehydration-responsive element binding (DREB) TFs by activation of biosynthesis (isopentenyl transferase, IPT) and degradation (dehydrogenase, CKX) CK genes. Whereas in the ABA direct CK regulation mechanism, ABA-responsive component like SNF1-related protein kinase 2 (SnRK2) can inhibit CK action by directly phosphorylating a negative regulator of CK signaling called type-A RR5 (ARR5) [83]. Drought stress can also induce a decrease of GA concentration in maize seedlings, thus increasing DELLA activity, which negatively regulate GA signaling, thus causing an increase of ROS quenching capacity and improved survival [84, 85]. The DELLA response seems be mediated by JA; in fact, the expression of the DELLA protein RGL3 increased JA signaling by an inhibiting interaction with jasmonate-ZIM domain (JAZ) proteins [86]. Verma et al. [73] have been supposed that DELLA and RBOH can have a pivotal role in the signaling/response pathway mediated by ABA, auxin, GA, JA and calcium fluxes under drought stress.

### Yield parameters and water use efficiency

**Fig. 6:**
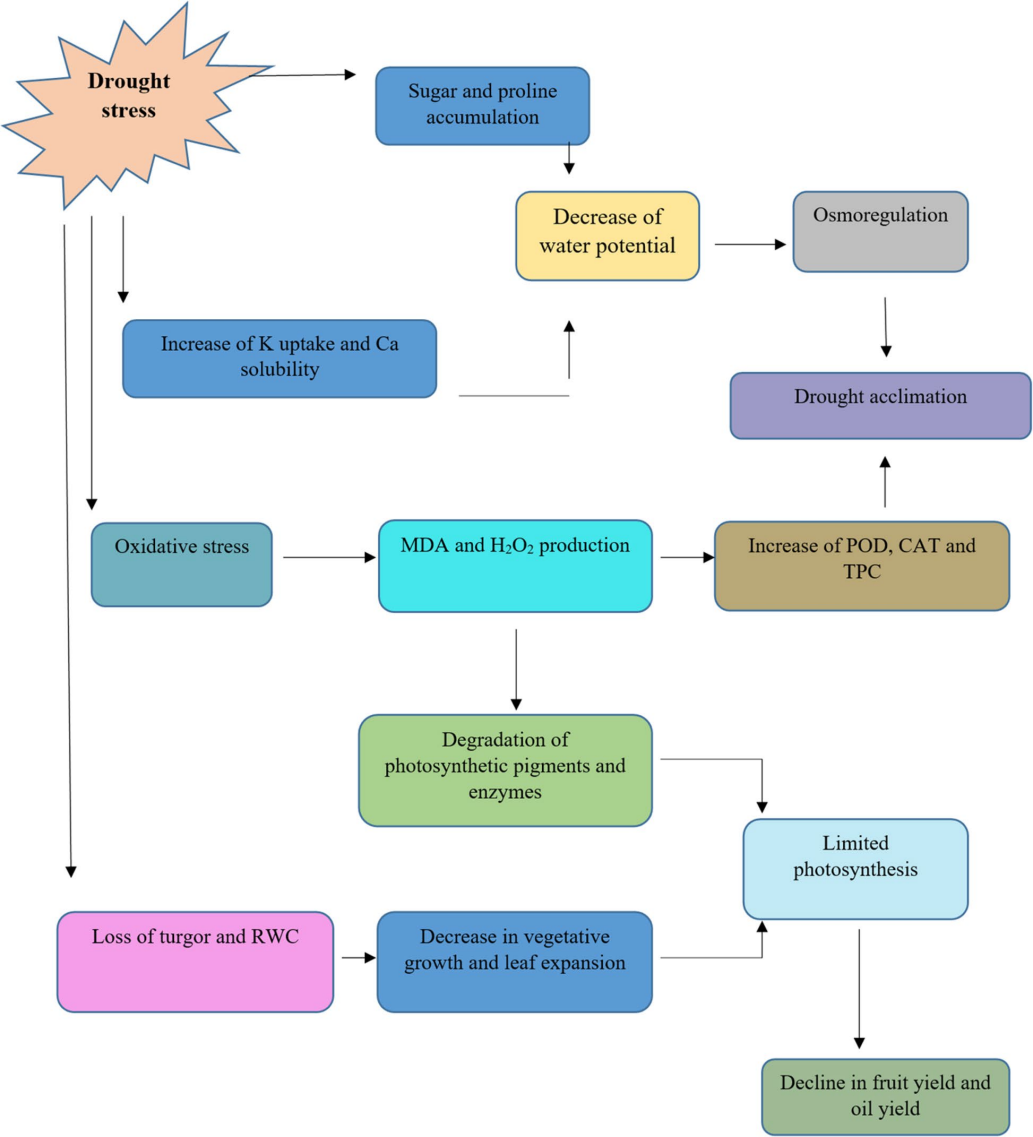
Possible mechanisms to enable the olive tree to drought tolerance and negative effects of drought stress on biochemical and yield parameters. MDA: Malondialdehyde; H_2_O_2_: Hydrogen peroxide; POD: Peroxidase; CAT: Catalase; TPC: Total Phenolic Content; RWC: Relative Water Content

According to our results, Konservolia demonstrated the highest fruit yield under full irrigation and the highest oil yield and fruit water use efficiency under 75% deficit irrigation. All the sensitive cultivars including Roughani, under full irrigation conditions showed a high fresh oil content. However, water use efficiency is one of the most important factors to evaluate yield and the management of water consumption of plant crops [67]. Drought could highly impair fruit set and subsequent lower yield due to the increased presence of imperfect flowers in olive. On the contrary, adequate irrigation during flower initiation and induction improved perfect flowers and, consequently, fruit yield in Shengeh cultivar of olive trees [87]. The reduction of fruit yield has been therefore associated with fruit moisture and irrigation level [88]. An experiment of [89], demonstrated that stem water potential was significantly correlated in olive cultivars with irrigation regime, oil yields were significantly greater and fruit yields were significantly lower in water-stressed trees (ψ_stem_ =  − 2.1 MPa) versus the un-stressed trees (ψ_stem_ =  − 1.6 MPa). It was also proved that oil percentages were significantly higher in stressed trees compared to control. Oil yield is impacted by oil percentage and fruit yield per hectare [29]. In fact, in an investigation, high oil yield in response to 100% full irrigation treatment was due to high fruit yield per hectare, but under 50% deficit irrigation treatment, oil yield was lower because of low fruit yield; generally, a water deficit harms fruit dry matter and oil accumulation [29]. In the direct mechanism, some genes might become active and enhance oil production while in the indirect one, drought stress decreases vegetative growth. The latter case determines more light penetration into the crown, and an acceleration of fruit ripening, both of which will improve oil content. Water scarcity induces morphological, physiological, and biochemical responses in sensitive plants responsible for the activation of a wide spectrum of metabolism changes to minimize osmotic and oxidative stress and reactivate photosynthesis and growth (Fig. 6). The increase of K^+^ and Ca^2+^ as well as of free sugars and proline (osmolytes) may play an important role in osmoregulation and drought acclimation. On the other hand, if drought-dependent oxidative stress is not overcome by reparative antioxidant systems (enzymes and metabolites), the strong increase in MDA and H_2_O_2_ proves that the oxidative/photooxidative stress has already caused photosynthetic dysfunction and leaf senescence, with a strong negative effect on developmental processes, plant growth, and fruit and oil yield.

## Conclusion

In our study, the presence of drought stress and mechanism of tolerance has been proven in almost all plants, nonetheless, the effects of both stress and tolerance responses are diverse from one cultivar to another. In particular, in response to restricted irrigation regimes, within the studied cultivars, Dezful and Konservolia are predicted as the best performers in the measured features. They displayed multiple adaptive mechanisms to counteract the harmful effect of water deficit, in particular, higher Ca and K and lower Na, higher Chls, compatible compounds, and enzymatic and non-enzymatic antioxidant activity, confirming their lower sensitivity to a rather high threshold of drought stress. Our results may provide useful indications in the selection of olive tree cultivars suitable for drought regions. Indeed, these selected cultivars can be useful for future more in-depth studies to unravel the molecular mechanisms of signal transduction (in particular related to hormonal crosstalk) underlying drought tolerance. However, they can also be used to design new agronomic strategies for olive cultivation to be translated directly in field to improve oil production even under stress conditions.

## Methods

### Experimental site, layout, and drought imposition

**Fig. 7:**
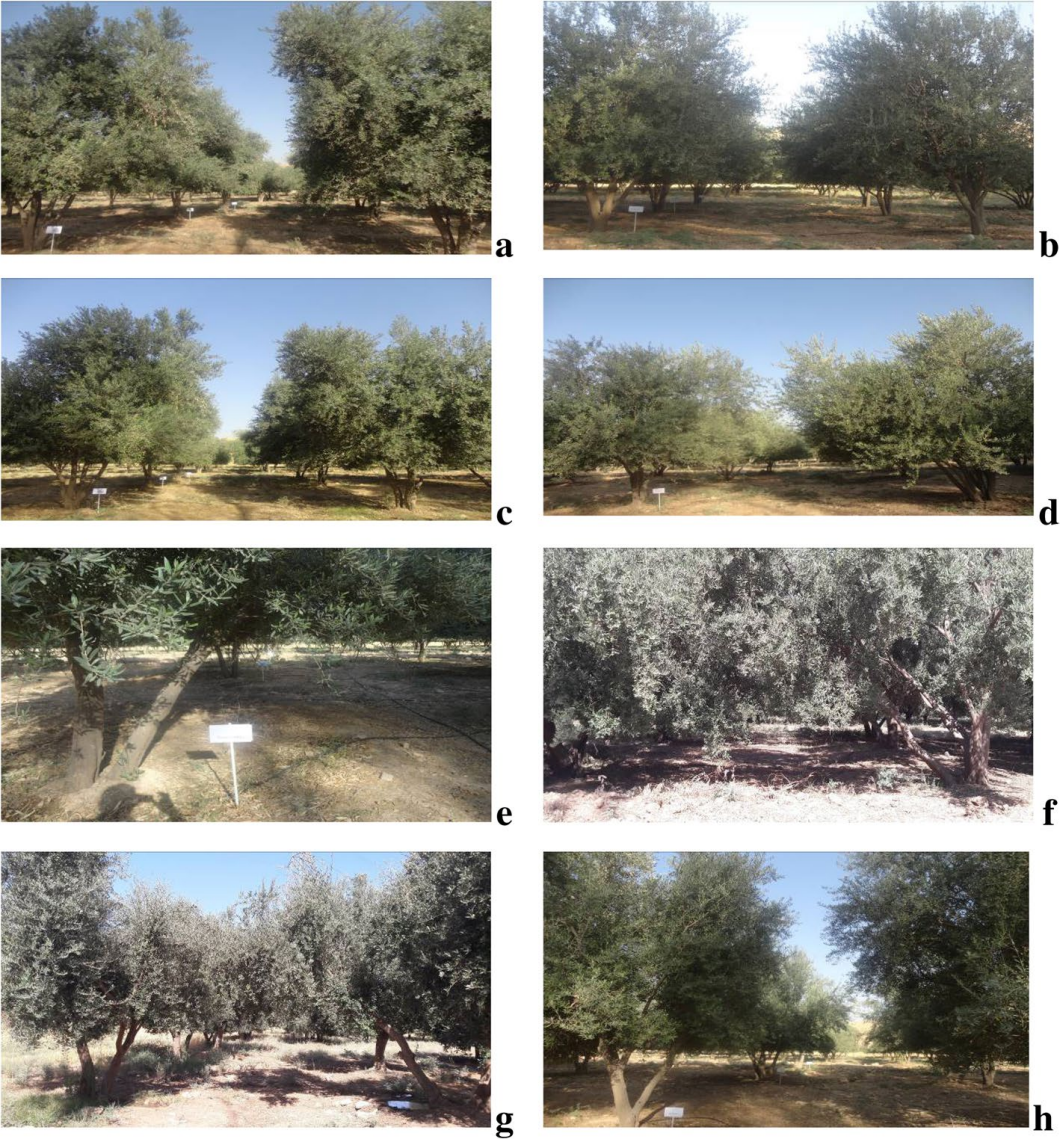
Different olive cultivars used in this study. Dezful (**a**), Roughani (**b**), Zard Aliabad (**c**), Shengeh (**d**) (local olive cultivars); and Konservolia (**e**), Sevillana (**f**), Manzanilla (**g**) and Mission (**h**) (foreign olive cultivars)

This experiment was accomplished in 2020 at Sarpole-Zahab in Dallaho Olive Research Station (34º30′ N, 45º51′ E, elevation above sea level: 581 m) in Kermanshah province, Iran. This study was conducted on 20-year-old olive trees and every experimental unit (every treatment) included two trees (olive trees planted in a frame of 6 m × 6 m). Four elite native olive cultivars (Zard Aliabad, Roughani, Dezful, and Shengeh) and 4 foreign olive cultivars (Manzanilla, Sevillana, Konservolia, and Mission) (Fig. 7) underwent 3 irrigation regimes. To characterize these cultivars deeply, refer to Table S1 [90]. Three treatment levels including 100% full irrigation, 75%, and 50% deficit irrigation were applied through the drip irrigation system simultaneously during the growing season. To calculate the water requirement, the FAO method was used, which was explained by Gholami et al. [9]. Irrigation of trees was done once every 3 days and total of 10 times per month. So that to irrigate the trees in each irrigation, the amount of water required for each tree was calculated according to climatic conditions, and stress levels, and the average irrigated water of each tree per month is shown in Table S2 and S3. The drought trial period lasted from August (after the last rain) until October. There was no rain at the experiment location during the study period. At the end of drought imposition, a sampling event took place in the autumn (at the end of the growth season). In Table S4, the average maximum and minimum temperature, relative humidity, and rainfall were presented.

### Growth parameters determination

Shoot and fruit length and diameter were determined with a vernier caliper. The fresh weight of the olive was measured by the digital scale at the termination of the drought stress period. Forty randomly selected fruits from every part of the olive trees of each replication were collected.

### Measurement of photosynthetic pigments

The amounts of Chls were measured according to the method of Lichtenthaler [91]. Fresh leaf tissues (100 mg) were sliced and extracted in 5 mL of 80% acetone in darkness. The absorption of the collected supernatant was measured with a spectrophotometer (Cary 100, Richmond, VA, USA) at the wavelengths of 646 and 663 nm. The following equations were used to estimate the amounts of Chl a, Chl b, and total Chl:

$$\mathrm{Chl a }({\mathrm{mg}.\mathrm{g}}^{-1}\mathrm{FW})= ({12.25\mathrm{A}}_{663}-{2.79\mathrm{A}}_{646})$$

$$\mathrm{Chl b }({\mathrm{mg}.\mathrm{g}}^{-1}\mathrm{FW})= ({21.21\mathrm{A}}_{646}-{5.1\mathrm{A}}_{663})$$

$$\mathrm{Total Chl }({\mathrm{mg}.\mathrm{g}}^{-1}\mathrm{FW})=\mathrm{ Chl a }+\mathrm{ Chl b}$$

### Osmolytes (soluble carbohydrates, proline, and TPC) and RWC

Fresh leaf tissue (0.5 g) was extracted in 10 mL of 70% ethanol. The extract was centrifuged at 2500 rpm for 5 min. Then, the upper phase of the samples was supplemented with 0.1 mL of anthrone. After thorough mixing, the absorbance was read at 625 nm using a spectrophotometer [92].

Approximately 0.5 g of leaf tissue was homogenized in 7.5 mL sulfuric acid (3%) to extract free proline (centrifuged at 10000 rpm for 15 min) which was mixed with glacial acetic acid and ninhydrin reagent based on the procedure of Bates [93]. Toluene (4 mL) was added before vortexing vigorously and the absorption was read at 520 nm.

Leaf tissue (2 g) was extracted with 10 mL methanol: distilled water (80:20) and 2 drops of tween-20. The solution was centrifuged at 10000 rpm for 20 min, after which the pellet was broken and re-suspended in the extract and centrifuged for another 10 min. Filtered extract was added to Folin-Ciocalteu’s phenol (5 mL) and sodium carbonate 15% (2 mL) and TPC was assayed by Slinkard and Singleton [94]. The resultant mixture was maintained in darkness for 2 h and absorption was read at 765 nm, and the content of total phenols was calculated by using the following relation:

Total phenol (mg kg^−1^) = Gallic acid (mg ml^−1^) × V(ml) × 1000/W(g).

Where V is the extraction volume and gallic acid concentration is established from the calibration curve.

RWC of the fully developed leaves was determined according to the method of Turner [95]. Further, RWC was calculated by the following equation:

$$\mathrm{RWC}\left(\mathrm{\%}\right)=100\times \frac{\mathbf{F}\mathbf{W}-\mathbf{D}\mathbf{W}}{\mathbf{T}\mathbf{W}-\mathbf{D}\mathbf{W}}$$

where FW is the fresh weight, DW is the dry weight of the leaves after drying in the oven at 75ºC for 72 h, and TW is the weight at full turgor when rehydration of the leaves occurred by floating on distilled water in the dark for 24 h.

### Mineral element measurements (Ca, K, and Na)

To determine the concentrations of calcium (Ca^2+^), potassium (K^+^), and sodium (Na^+^) ions in the aerial organs and fruits, oven-dried plant materials were first digested by nitric acid and H_2_O_2_ [96]. The concentration of Ca was measured using a flame atomic absorption spectrophotometry (Perkin Elmer, San Francisco, CA, USA), while the K and Na concentrations were determined using a PFP7 industrial flame photometer (Jenway Tech., Stone, Staffordshire, UK).

### Oxidative enzymes (CAT and POD) and marker (MDA)

CAT activity was assayed using the Aebi [97] method. One unit of CAT activity was defined as the degradation of H_2_O_2_ per minute by using the extinction coefficient of 40 mM^−1^ cm^−1^ at 240 nm. The reaction mixture included 3 mL phosphate buffer pH 7.0 50 mM, 1 mL enzyme extract, and 5 µl H_2_O_2_ (30%).

POD activity was determined according to the method of Nakano and Asada [98], using the absorption changes of a reaction mixture (3 mL potassium phosphate buffer 50 mM, 4.51 µl H_2_O_2_ 30%, enzyme extract). The absorption was read at 290 nm after 2 min of incubation.

MDA content was quantified by the method of Heath and Packer [99]. 200 mg of leaves were extracted in 1% trichloroacetic acid (5 mL) followed by centrifugation at 15,000 rpm for 15 min. Then 4 mL of trichloroacetic acid (20%) with thiobarbituric acid (0.5%) was added and the mixture was heated in a water bath (95ºC for 30 min). MDA content was calculated by the following relation:

$$\mathrm{A}=\mathrm{EBC}$$

where A is the absorption at 600 nm, E is the extinction coefficient (155 mM^−1^ cm^−1^), B, the cuvette width and C is the MDA content (mM).

### Oil content and water use efficiency

Olive oil extraction was carried out using the I.O.O.C (2002) protocol. The pitted fresh fruits were dried for 48 h at 70 ºC. Dried samples (2 g) were then subjected to Soxhlet extraction with diethyl ether (250 mL) for 5 h followed by the transfer into an oven for 2 h drying at 70 ºC. The mass difference between the sample weights during two consecutive dryings is considered as the oil content in the dry matter. To determine the oil content in fresh fruit flesh, the value of oil content in the dry matter should be multiplied by the fruit dry matter percentage.

Water use efficiency (or crop water productivity) was calculated for each plant by dividing total dry matter production (kg ha^−1^) by the cumulative amount of water used (m^3^ ha^−1^) during the growing season [9].

### Statistical analysis

The experiment was carried out as a Randomized Complete Block Design (RCBD) in a factorial arrangement with 24 treatments (8 cultivars and 3 irrigation regimes) and each experimental unit was provided with 3 replicates (Each experimental unit included two trees in 3 replicates; 2 × 3 = 6 trees). The statistical analysis and computations were performed using SAS (v.9.1) and least significant difference (LSD) test to determine whether the mean values were significantly different at the *Ρ* ≤ 0.05 level.

## Supplementary Information

**Additionalfile 1: Table S1. **Main characteristicsof native and foreign olive cultivars undegoing water shortage in the Dallaho Olive Research Station in Sarpol-Zahab city, Kermanshah Province. **Table S2.** Estimation of required irrigation water volume basedon evapotranspiration in 2020. **Table** **S3.** Estimation of irrigation water volume required in different irrigationtreatments in 2020. **Table S4. **Average monthly temperature, relative humidity, evaporation and rainfall ofSarpol-e zahab (2020).

## Data Availability

The datasets generated and/or analyzed during the current study are available from the corresponding author on reasonable request.

## Abbreviations

POD: Peroxidase
CAT: Catalase
SOD: Superoxide dismutase
RWC: Relative Water Content
ROS: Reactive Oxygen Species
MDA: Malondialdehyde
H_2_O_2_: Hydrogen peroxide
Chl: Chlorophyll
FW: Fresh Weight
DW: Dry Weight
Ca: Calcium
Na: Sodium
K: Potassium
