# Supplementary material for: Effect of three water-regimes on morpho-physiological, biochemical and yield responses of local and foreign olive cultivars under field conditions
Source: BMC Plant Biol. 2022 Oct 7;22:477. doi: 10.1186/s12870-022-03855-8 (PMC9540738; doi:10.1186/s12870-022-03855-8)
Supplement: Supplementary file 1 — Additionalfile 1: Table S1. Main characteristicsof native and foreign olive cultivars undegoing water shortage in the Dallaho Olive Research Station in Sarpol-Zahab city, Kermanshah Province. Table S2. Estimation of required irrigation water volume basedon evapotranspiration in 2020. Table S3. Estimation of irrigation water volume required in different irrigationtreatments in 2020. Table S4. Average monthly temperature, relative humidity, evaporation and rainfall ofSarpol-e zahab (2020). [file 12870_2022_3855_MOESM1_ESM.doc]

**Table S1. Main characteristics of native and foreign olive cultivars undegoing water shortage in the Dallaho Olive Research Station in Sarpol-Zahab city, Kermanshah Province**

| **Cultivar** | **Original country** | **Commercial use** | **Fruit ripening time** | **Growth characteristic** |
| --- | --- | --- | --- | --- |
| Manzanilla | Spain | conserved | Late November | -Wide-spreading growth habit  -high canopy density |
| Sevillana | Spain | conserved | Early December | -stand growth habit  -moderate canopy density |
| Mission | America | Dual purpose | Early December | -stand growth habit  -high canopy density |
| Konservolia | Greece | conserved | Mid October | -stand growth habit  -moderate canopy density |
| Zard Aliabad | Iran | Dual purpose | Mid October | Wide-spreading growth habit  -high canopy density |
| Roughani | Iran | Oil production | Early October | -stand growth habit  -moderate canopy density |
| Dezful | Iran | conserved | Mid October | -stand growth habit  -moderate canopy density |
| Shengeh | Iran | conserved | Mid October | -Wide-spreading growth habit  -high canopy density |

**Table S2.** Estimation of required irrigation water volume based on evapotranspiration in 2020

| **Month** | **Evapotranspiration (mm)** | **Monthly water requirement**  **(L/tree)** | **Monthly water requirement**  **(m^3^/ha)** |
| --- | --- | --- | --- |
| May | 258.4 | 4428/53 | 1230.11 |
| Jun | 343.7 | 6411.13 | 1780.81 |
| July | 374.9 | 7353.29 | 2042.52 |
| Aug | 356.7 | 6960.34 | 1933.37 |
| Sep | 317.1 | 6179.66 | 1716.52 |
| Oct | 228.7 | 4485.72 | 1245.99 |
| Sum total | 1879.5 | 35818.70 | 9949.36 |

**Table** **S3.** Estimation of irrigation water volume required in different irrigation treatments in 2020

| **Treatment** | **Monthly water requirement**  **(lit /tree)** | **Monthly water requirement**  **(m^3^/ha)** |
| --- | --- | --- |
| 100% water requirement | 35818.70 | 9949.36 |
| 75% water requirement | 26864.02 | 7462.01 |
| 50% water requirement | 17909.35 | 4974.68 |

**Table S4.** Average monthly temperature, relative humidity, evaporation and rainfall of Sarpol-e zahab (2020)

| **Month** | **Maximum temperature (°C)** | **Minimum temperature**  **(°C)** | **Minimum relative humidity (%)** | **Maximum relative humidity (%)** | **Rainfall (mm)** |
| --- | --- | --- | --- | --- | --- |
| May | 31.5 | 14.2 | 24 | 65 | 1 |
| Jun | 38.8 | 18.4 | 12 | 42 | 0 |
| July | 42.2 | 23.5 | 12 | 36 | 0 |
| Aug | 42.8 | 24.2 | 14 | 38 | 0 |
| Sep | 41 | 21.2 | 13 | 38 | 1 |
| Oct | 33.6 | 11 | 10 | 37 | 0 |
